# A COVID-19 Rehabilitation Prospective Surveillance Model for Use by Physiotherapists

**DOI:** 10.3390/jcm10081691

**Published:** 2021-04-14

**Authors:** Paula Postigo-Martin, Irene Cantarero-Villanueva, Ana Lista-Paz, Eduardo Castro-Martín, Manuel Arroyo-Morales, Jesús Seco-Calvo

**Affiliations:** 1 Health Sciences Faculty, University of Granada, 18016 Granada, Spain; paulapostigo@ugr.es (P.P.-M.); eduardoc@ugr.es (E.C.-M.); marroyo@ugr.es (M.A.-M.); 2 Sport and Health Research Center (IMUDs), 18016 Granada, Spain; 3 Instituto de Investigación Biosanitaria ibs. GRANADA, 18014 Granada, Spain; 4 Unit of Excellence on Exercise and Health (UCEES), University of Granada, 18016 Granada, Spain; 5 Faculty of Physiotherapy, University of La Coruña, 15006 La Coruña, Spain; ana.lista@udc.es; 6 Physiotherapy Department, Institute of Biomedicine (IBIOMED), University of Leon, Campus de Vegazana s/n, 24071 Leon, Spain; dr.seco.jesus@gmail.com; 7 Department of Physiology, Visiting Professor and Researcher of University of the Basque Country, 48940 Leioa, Spain

**Keywords:** cardiorespiratory system, COVID-19, mental health, neuromuscular system, prospective surveillance model

## Abstract

The long-term sequelae of coronavirus disease 2019 (COVID-19) are only now beginning to be defined, but it is already known that the disease can have direct and indirect impacts mainly on the cardiorespiratory and neuromuscular systems and may affect mental health. A role for rehabilitation professionals from all disciplines in addressing COVID-19 sequelae is recognised, but it is essential that patient assessment be systematic if health complications are to be identified and treated and, if possible, prevented. The aim is to present a COVID-19 prospective surveillance model based on sensitive and easily used assessment tools, which is urgently required. Following the Oxford Centre for Evidence-Based Medicine Level of Evidence Tool, an expert team in cardiorespiratory, neuromuscular and mental health worked via telemeetings to establish a model that provides guidelines to rehabilitation professionals working with patients who require rehabilitation after suffering from COVID-19. A COVID-19 prospective surveillance model is proposed for use by rehabilitation professionals and includes both face-to-face and telematic monitoring components. This model should facilitate the early identification and management of long-term COVID-19 sequelae, thus responding to an arising need.

## 1. Introduction

Multisystem long-term sequelae are being described following severe acute respiratory syndrome coronavirus 2 (SARS-CoV-2) infection, with a high impact at cardiorespiratory, neuromuscular [1] and psychological levels [1,2] and possibly other systems [3]. The latest reports have established that 26% of patients needed hospitalisation, and among them, 14% required an intensive care unit (ICU) admission and/or respiratory support [4]. The long-term sequelae of the infection are now beginning to be defined; many may seriously impair the health of individuals and have important knock-on economic effects [5]. Given the similarities between coronavirus disease 2019 (COVID-19) and diseases caused by other coronaviruses [6], post-acute and long-term sequelae that require specific treatment should be expected [1,7]. The number of people eventually requiring attention for COVID-19 sequelae could be high: on 30 January 2021, the present pandemic had already affected 102,107,858 people worldwide [8], being the main worldwide health problem with an uncertain future.

**Figure 1:**
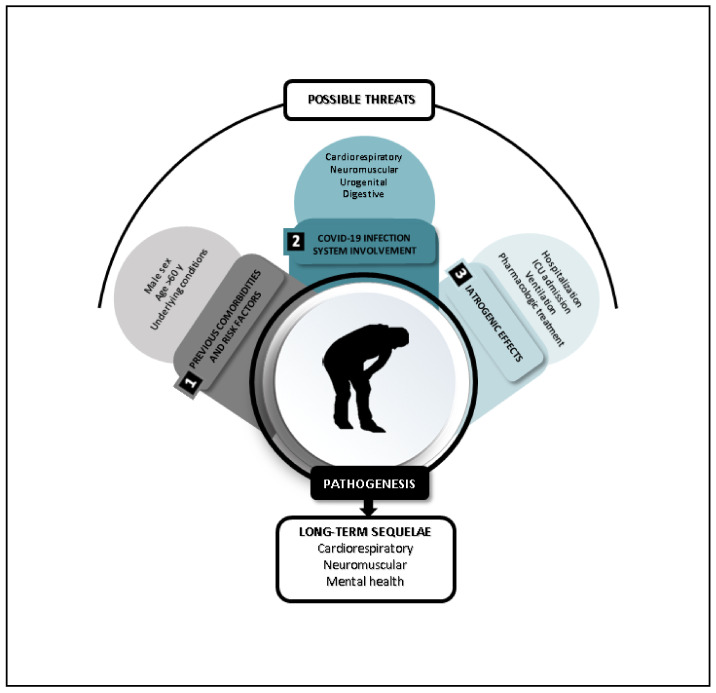
Profile of patients likely to require rehabilitation after coronavirus disease 2019 (COVID-19).

Recent data show that individuals with a severe presentation of the illness have underlying comorbidities such as hypertension, diabetes, cardiovascular disease (CVD) or cerebrovascular disease [9], and hospitalised patients require a long hospital (around 4–53 days) or ICU (4–19 days) stay [10]. In addition, it must be considered that these patients may suffer from the iatrogenic effects of COVID-19 treatment [11] (Figure 1). Together, these factors render them vulnerable to loss of function and reduced quality of life after release from the hospital [6]. Most of these health issues can be at least partly treated by rehabilitation professionals (RPs) from all disciplines. Indeed, a role for the RP in addressing these sequelae is recognised [12,13], but it is essential that patient assessment be systematic if further health complications are to be identified, treated and, if possible, prevented. Clear guidelines are therefore required (1) to help RPs evaluate the likelihood of a patient experiencing major health deterioration and (2) assist them in providing appropriate treatment. Some previous studies [14,15] have presented successful models for assistance in early detection of health-related issues and the ability to take fast actions. In ICU-admitted patients, a real-time surveillance model was used to predict acute respiratory distress syndrome (ARDS) prior to its onset, with multiple, non-invasive and easy-to-collect vital signs, related to the risk factors that associate with ARDS, and easy to translate to home surveillance. This model was found to outperform the classical tests [14]. Another example in ICU-admitted patients is a model that successfully predicted ARDS events to support early diagnosis and intervention to improve survival rates [15]. In patients with cancer, several similar prospective surveillance models (PSMs) for physiotherapy interventions have been developed [16,17,18,19]. These previous experiences led us to suggest that the implementation of this COVID-19 PSM would be feasible [18], may identify early impairment [18,19,20], predict the severity of possible impairments [16,17], allow early intervention [19,21,22] and potentially save resources [22]. 

Currently, to the best of our knowledge, there are four models for COVID-19 [23,24,25]. These models successfully aim to optimise resource expenses in preoperative tests, while considering personal safety [23]; preserve resources of ambulatory service [24]; predict high-risk patients with admission data [25]; and a multidisciplinary model that aimed to detect patients with increased physical and mental health care needs [26]. However, considering the possible long-term sequelae of COVID-19 [27] and long-COVID-19 with persistent symptoms [28,29], we have not found any model that focusses on monitoring these impairments, with follow-up periods, after COVID-19 and focusses on impairments approachable from physiotherapy. Therefore, considering previous models, we propose a PSM for early detection that allows early intervention by physiotherapists with the aim to improve current health assistance.

The present work proposes a prospective surveillance model (PSM), based on sensitive and easily used assessment tools, for use by RPs when treating patients who require rehabilitation after suffering from COVID-19. Importantly, this PSM contemplates face-to-face and telematic patient monitoring.

## 2. Methodology

A panel of RPs (I.C.-V., M.A.-M., J.S.-C., A.L.-P., E.C.-M. and P.P.-M.), the members of which are experts in alterations of functionality (I.C.-V., M.A.-M. and J.S.-C.), the cardiorespiratory system (M.A.-M. and A.L.-P.), the neuromuscular system (E.C.-M.) and mental health (E.C.-M. and P.P.-M.), was brought together to prepare the proposed COVID-19 prospective surveillance model (COVID19-PSM). The process began at the end of March after detecting the urgent need to offer RP support beyond acute care for the disease. After initial contact, it was decided that the Oxford Centre for Evidence-Based Medicine Level of Evidence Tool [30] will be used to establish decision-making. After the selection of panel members (performed by I.C.-V. and M.A.-M.), the following tasks were undertaken by those with the corresponding expertise: (1) a review of the epidemiological literature (P.P.-M.) and (2) a review of the available information regarding the involvement of, and the sequelae affecting, the cardiovascular (I.C.-V. and M.A.-M.), respiratory (A.L.-P.), neurological (E.C.-M.) and musculoskeletal (I.C.-V., M.A.-M. and J.S.-C.) systems, as well as the mental health problems associated with COVID-19 (E.C.-M.). The latter reviews included the clinical assessment tools available for identifying risk thresholds and treatment options. Information was also sought about the main components of PSMs (Table 1). These tasks involved searches of Medline (PubMed), the Web of Science, Medrxiv and official resources (e.g., documents published by the World Health Organisation -WHO- and the Centers for Disease Control and Prevention -CDC-). Search terms were modified to fit each database. No language restriction was enforced.

Each expert assessed the quality of the information available in their field using the Oxford Centre for Evidence-Based Medicine (OCEBM) 2009 Level of Evidence Tool (see Supplementary Materials S1) and sent a final report to the lead author (I.C.-V.). These reports were then organised into a single document, which was distributed to all panel members for comprehensive study. Telemeetings were then held to discuss the desirability of including the collected information in the proposed PSM. Decisions were taken by voting; ≥70% consensus was required for all inclusion/exclusion decisions. In total, three telemeetings (held between March 30th and 15th June 2020) were needed to define the contents of the PSM.

## 3. The Proposed Prospective Surveillance Model

**Figure 2:**
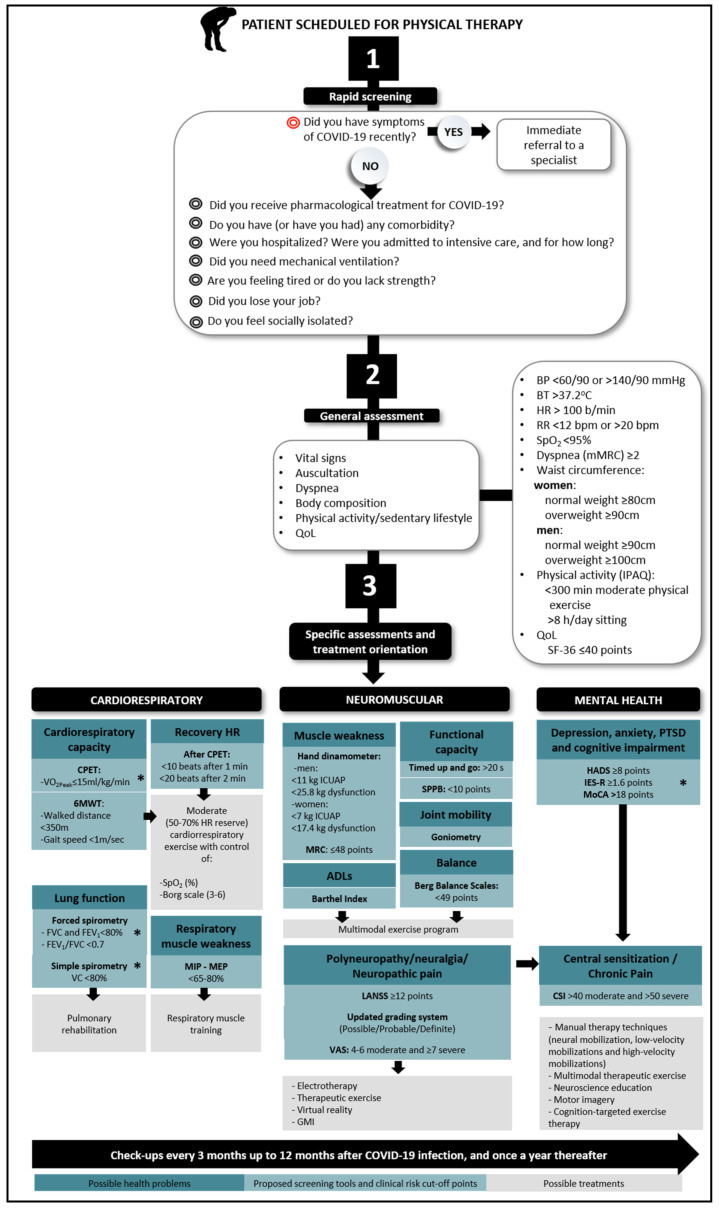
The proposed COVID-19 prospective surveillance model for use by physiotherapists. ADLs: activities of daily living; BP: blood pressure; BT: body temperature; CPET: cardiopulmonary exercise testing; CSI: central sensitization inventory; FEV1: forced expiratory volume; FVC: forced vital capacity; GMI: graded motor imagery; HADS: hospital anxiety and depression scale; HR: heart rate; ICU: intensive care unit; ICUAP: ICU-acquired paresis; IES-R: Impact of events scale-revised; IPAQ: international physical activity questionnaire; LANSS: Leeds Assessment of Neuropathic Symptoms and Signs; MEP: maximum expiratory pressure; MIP: maximal inspiratory pressure; mMRC: modified medical research council; MoCA: Montreal cognitive assessment; MRC: Medical Research Council Scale; PTSD: post-traumatic stress disorder; QoL: quality of life; RR: respiratory rate; SF-36; The Short Form-36 Health Survey; SpO2: peripheral capillary oxygen saturation; SPPB: short physical performance battery; VAS: visual analogue scale; VC: vital capacity; Vo2peak: peak rate of oxygen uptake; 6MWT: 6 minute walking test; *: refer to specialist.

The proposed COVID19-PSM for use by RPs (Figure 2) is divided into three sections: rapid screening, general assessment and specific assessments for each system likely to be affected. The latter section includes reliable tools for making necessary assessments, cut-off points and orientation regarding treatment.

### 3.1. Rapid Screening

The possibility that a patient has been re-infected with SARS-CoV-2 needs to be quickly ruled out. This can be done via the screening questions [31] shown in Figure 2. Re-infection is a red flag that demands immediate referral to a specialist; patients with active COVID-19 should not be treated using the proposed PSM as a guide.

Once re-infection has been ruled out, anamnesis can be performed to detect further red flags [32] and grey flags regarding sequelae. The term grey flag is here proposed to describe findings that need to be borne in mind by the RP, since they may complicate recovery and accelerate the possibility of a process becoming chronic, but that do not require immediate referral to a specialist. Such grey flags may reflect factors related to comorbidities, the treatment received for COVID-19 and biopsychosocial and work-related matters. Anamnesis can be performed by phone or via the internet [7] and should be whenever possible.

### 3.2. General Assessment

This includes a number of simple assessments to detect health concerns that should indicate any condition that might be driving the emergence or perpetuation of more serious health issues (Figure 2). The results provide information regarding the need for more specific assessments and help in the design of intervention strategies. It should be remembered that COVID-19 commonly affects persons with a sedentary lifestyle and reduced physical activity [33], who are usually overweight and/or who have hypertension, cardiovascular disease and/or diabetes [9]. It may well be that after suffering from COVID-19, patients move even less than before [34]. This can facilitate the appearance of comorbidities or accentuate those already present [35]. RPs should therefore encourage salutogenesis in their patients [36]. Online technologies, which have already been used in other health settings for this purpose [7], might be adapted for use with survivors of COVID-19.

### 3.3. Specific Assessments

#### 3.3.1. Cardiorespiratory System

Based on the effects of severe acute respiratory syndrome (SARS) and Middle East respiratory syndrome (MERS) [6], both caused by coronaviruses, long-term cardiac and pulmonary sequelae might be expected to develop after suffering from COVID-19 [37]. Indeed, some 32.8–70% of patients hospitalised because of COVID-19 develop ARDS [38], and some 20% of these are known to suffer cardiac sequelae [39]. For instance, having a history of stroke increases the risk of death from COVID-19 by up to three times [40], and out of those patients with a severe presentation, 5.7% developed cerebrovascular complications [41]. It has also been described that following post-intensive care syndrome (PICS), patients may show reduced pulmonary function, weakness of the respiratory muscles [32] and cardiovascular complications (e.g., myocardial injury) [42] that might persist for months and possibly even years. Thus, specific cardiorespiratory assessments (Figure 2) should be made frequently, and the patient referred to a specialist if there is any suspicion of an abnormality (e.g., an altered spirometry pattern, hypertension, high resting heart rate, thrombotic events, etc.).

#### 3.3.2. Neuromuscular System

Around 20% [43] of patients who develop severe or critical clinical COVID-19 are at risk of neuromuscular complications [44], such as critical illness polyneuropathy or critical illness myopathy (CIP or CIM, respectively). Certainly, 25–68% of patients with ARDS who have been admitted to an intensive care unit for a long period develop CIP or CIM [45]. The same might therefore be expected in patients with severe COVID-19 who may also spend a long time in intensive care [38]. Neurological sequelae have also been described for both SARS and MERS [46,47]. These complications affect the patients’ general functional capacity, making it difficult for those affected to perform normal activities of daily living or to return to work [32] (which entails economic problems). The proposed COVID19-PSM includes common quantitative tests for muscle weakness, functional capacity, joint mobility, balance, polyneuropathy, neuralgia and neuropathic pain able to identify possible long-term sequelae (Figure 2). The systematisation of these assessments should allow for early identification and appropriate intervention and help decide on the need for referral to other health professionals.

#### 3.3.3. Mental Health

Mental health can be compromised in survivors of ARDS [48] and PICS [32]. For example, at discharge from intensive care, 70–100% of patients with ARDS may show cognitive decline [49], which may be long term. Indeed, 46–80% of these patients remain affected at one year and 30% at five years [49]. It is already becoming clear that COVID-19 survivors who required intensive care are at risk of such long-term problems [2], but they may also suffer depression, anxiety and post-traumatic stress disorder [32]. These conditions are believed to compromise the immune system and may complicate recovery [50]. This COVID19-PSM therefore includes mental health assessments to detect a range of problems (Figure 2). Patients identified as suffering from mental health problems should be referred to an appropriate specialist.

## 4. Discussion

This study presents a PSM for the early detection of long-term COVID-19 sequelae to adopt preventive strategies and propose treatment measures. In physiotherapy, there are not many studies that have addressed problems this way, although there are some important PSMs in a population such as cancer. Regarding diagnosis and prevention, the implementation of these previous models has allowed researchers to detect early impairments or their severity and make an early intervention [16,17,18,19]. The same occurs in our PSM, which aims to address COVID-19 or long-COVID-19 sequelae and side effects derived from its management in the ICU and benefits from an early stage and suggest intervention. In addition, there is already a model in COVID-19 [26] that detects patients with increased physical and mental health care needs. However, that model is multidisciplinary, and the one we presented is centred in physiotherapy as there are many COVID-19-related sequelae that physiotherapists can manage. Regarding cost-effectiveness, previous models have been shown to potentially save resources [19], yet we do not know whether our PSM will be cost-effective, and if effective, it will save the costs derived from impairments in a long-term period by avoiding them from the earliest moment.

The key point of this PSM is the suggestion of the use of affordable instruments capable of detecting early impairments on the main systems affected by COVID-19: (1) at the first evaluation, with rapid screening through exploratory questions; (2) at general health assessment (vital signs, auscultation, dyspnoea, body composition, physical activity level, sedentary lifestyle and quality of life); and (3) at specific evaluation of cardiorespiratory, neuromuscular and mental levels (Figure 2). It should be noted that the PSM allows the detection of health problems that require referral to specialists (marked with an asterisk in Figure 2); it being a disease that affects multiple systems, with varying degrees of involvement, management with multidisciplinary groups is the optimal decision.

The questions that appear in the rapid screening (Figure 2, Number 1) have been used for the detection of PICS [32] and musculoskeletal disorder [51] complications in adults, and its inclusion within the evaluation and treatment processes has shown to improve outcomes in physiotherapy units [52]. Currently, there is a fundamental question oriented to detect a person with an active COVID-19 illness (Did you have symptoms of COVID-19 recently?), our red flag, that must precede any assessment [31]. After this, the following proposed questions try to deepen the situation experienced by each patient; they should be considered as they can influence correct rehabilitation, but they do not require immediate referral.

The general assessment (Figure 2, Number 2) aims to have an overall picture of the health status of patients, for which it establishes cut-off points stated for COVID-19 [53], international guidelines [53,54] or validation studies [55]. These cut-off points serve for identifying potential health risks before starting rehabilitation and should be considered as exclusion criteria for safety. These gather values of blood pressure (BP) of <90/60 or >140/90 mmHg, a heart rate (HR) of >100 beats/min, peripheral capillary oxygen saturation (SpO_2_) of <95% [53], a respiratory rate (RR) of <12 or >20 bpm [55], a body temperature (BT) of >37.2 °C or dyspnoea of ≥2 grade in the modified Medical Research Council (mMRC) scale [54].

Another parameter to consider is the waist circumference, which is becoming more important and even has been considered by some authors as a vital sign [56] due to its important relationship with morbidity [57] and cardiovascular death [58]. Regarding this, the WHO established values of ≥90 cm in women and ≥100 cm in men as cut-off points of higher risks for heart disease, diabetes and stroke [59]. Waist circumference is a surrogate measure of visceral fat [60] that has been linked to being present in patients with higher COVID-19 severity [61] and a stronger need for intensive care [62], who usually are more physically inactive [63] and sedentary [64], and these numbers have risen during the pandemic [65], favouring, therefore, weight gain [66]. This can be easily assessed [67]. In fact, already up to 47.7% of the worldwide population [68] and up to 72% in Europe were physically inactive and 23% were sedentary (with >10 h sitting per day). In addition, it was calculated that 9% were both physically inactive and sedentary [69]. Moreover, emerging evidence during the pandemic is showing that maintaining physical exercise practice outwards or inwards decreases COVID-19 severity [70]. Recommended levels of physical activity [71] and cut-off points [67,72] during the pandemic are only for the general population, but it is proposed that COVID-19 survivors regain physical activity with a safe stratify strategy [73]. A study stated that young and middle-aged adults who are moderately and/or severely affected usually have hypertension and overweight and suffer a significant impact on their quality of life [26]. It is therefore important to include a quality-of-life assessment [74] to check patients’ perception of their own physical and psychological well-being [75], which is already known to suffer from a reduction in all spheres [76].

To perform a specific evaluation (Figure 2,), it is important to follow a hierarchy in system impairments, going through health indicators with solid scientific evidence, which may be transferable to COVID-19 or long-COVID-19 survivors. It is important to measure the integrity of cardiopulmonary function, whose importance has increased due to COVID-19’s effects on this system, with impairments of 26% mid-term [76] to 46% long-term COVID-19 sequelae [77]. A recent study [76] showed a reduction in the maximal oxygen consumption and in the walked distance in the 6 min walking test (6MWT) after 2–3 months of moderate–severe COVID-19. Impairment has been considered in other population as values of peak oxygen consumption (VO_2_peak) of ≤15 mL/kg/min with a cardiopulmonary exercise test (CPET) [78], or <350 m with the 6MWT [79], or a gait speed <1m/s (REF). The CPET should be performed in situations of medical control, with exhaustive monitoring [80,81,82], considered the gold standard [83], and allows the detection of problems that should be assessed by a specialist. Although it could be too demanding, the 6MWT is an optimal alternative to use, both the CPET and 6MWT are already used in COVID-19 patients [84,85]. When an impairment is found, cardiovascular exercise of moderate intensity [32], with control of SpO_2_ (to stop if values decrease by <90% or a decrease by more than 4% from baseline [86]) and rate of perceived exertion in the Borg scale between 3 and 5 [87], is recommended for optimal outcome and safety [32]. Another parameter of interest is the recovery HR, which reflects the reactivation of the parasympathetic system after physical exercise, and it is implicated in cardiovascular risk events and all-cause mortality [88]. Monitoring objective fitness data is helpful to control the exercise tolerance in COVID-19 patients [73]. These data have been found deficient if the difference from the maximal achieved during physical exercise [89] is <10 or <20 after one and two minutes, respectively [88]. We believe that this indicator should be included as it is a simple and affordable measure to check cardiovascular issues related to exercise tolerance [88].

Long-term pulmonary impairments are present in up to 29% of COVID-19 survivors [27]. The key parameters regarding lung function are forced spirometry and simple spirometry as they have been set as the main methods to perform a respiratory function assessment [90]. The parameters to be considered as abnormal functions are forced vital capacity (FVC) and forced expiratory volume (FEV1) of <80% and FEV1/FVC of <0.7 [90]. These assessments should be complemented by respiratory muscle assessment with maximal inspiratory pressure (MIP) and maximum expiratory pressure (MEP) as the specific outcomes [91], where values of <65–80% for the MIP or MEP are considered pathological values [92]. Respiratory physiotherapy plays a crucial role in COVID-19 rehabilitation [93], and there are very specific techniques from Physiotherapy such as pulmonary rehabilitation [37,94] and respiratory muscle training [95], with evidence that they are useful in the management of this situation. It is recommended that physiotherapists consider a referral to specialists if important impairments are found.

A wide variety of affectations at a neuromuscular level have been described in COVID-19 survivors, such as facial paresis, Guillain-Barré syndrome, neuropathies, CIP and CIM, myalgia and myositis, among others [96], affecting functional capacity and activities of daily living [32]. For that reason, it is important to assess all parameters that help in detecting major problems [38,44,46]. In particular, due to the prolonged rest situation, muscle weakness, ambulation and balance must be assessed. These assessments are muscle weakness with grip strength (with cut-off points of dysfunction of <25.8 [97] and <11 kg [97] in men and <17.4 [98] and <7 kg [98] in women, being the last measure from ICU-acquired paresis) and the MRC scale of ≤48 points [99]. The activities of daily living might be affected as a consequence [32,47], so an assessment with the Barthel Index should be performed [100]. In addition, functional capacity should be assessed with the timed-up-and-go cut-off point of >20 s [101] and the short physical performance battery of <10 points [102], joint mobility with a goniometry assessment [103] and balance with the Berg Balance Scale and a cut-off of <49 points [104]. For polyneuropathy, neuralgia and neuropathic pain, a cut-off of ≥12 points is suggested in the Leeds Assessment of Neuropathic Symptoms and Signs (LANSS) scale [105]; the updated grading system and the grading of possible, probable or definite result [106]; and a visual analogue scale (VAS) of 4–6 as moderate or ≥7 as severe pain [107]. For these alterations, a multimodal therapeutic exercise [108] programme that includes aerobic, strength, stretching and balance has already been effective [109]. In fact, an aerobic programme has already been recommended [110], and strength programmes are already being implemented to assess muscle weakness in COVID-19 survivors [111]. For polyneuropathy, neuralgia and neuropathic pain, an intervention of electrotherapy [112], therapeutic exercise, virtual reality and graded motor imagery (GMI) [113] is also suggested, which has been effective in other populations, as there is little information about most neuromuscular alterations in COVID-19 survivors [114].

That these alterations may facilitate or be related to chronic pain [114] or an underlying central sensitisation must be considered as well [115]. For efficient detection of central sensitisation, the use of the central sensitisation inventory (CSI) has been recommended, where values of >40 or >50 points indicate moderate or severe central sensitisation based on comorbid symptoms and conditions of other central sensitisation syndromes [116]. For this purpose, physiotherapy has many techniques to include in the intervention, such as manual therapy (neural mobilisation, low-velocity mobilisation and high-velocity mobilisation) [117], multimodal therapeutic exercise [32], neuroscience education [117], motor imagery and cognition-targeted exercise therapy [118] used in patients with nociceptive or chronic pain. Moreover, chronic pain and central sensitisation form a close cluster, together with anxiety and depression [119]; therefore, they also must be addressed. Some studies have shown the prevalence of psychopathology and psychiatric sequelae in COVID-19 survivors, finding a presentation of 42% for anxiety, 31% for depression, 28% for post-traumatic stress disorder and 20% for obsessive-compulsive symptoms 1 month after hospital admission [120]. There are few data in a long-term period, but there have been findings of cognitive impairment 3 months post-discharge [121], so they may be present later as well if not addressed, as has happened in other coronavirus outbreaks with months to years of impairment [122]. From the perspective of physiotherapy, it is important to address and consider these alterations in our intervention. However, facing a well-established alteration involving depression, anxiety, post-traumatic stress disorder and cognitive impairments, a cut-off of ≥8 points in the Hospital Anxiety and Depression Scale (HADS) [123], ≥1.6 on the Impact of Events Scale—Revised [124] and/or >18 points on the Montreal Cognitive Assessment (MoCA) scale [125], respectively, has been proposed to consider a referral to a specialist.

### Limitations, Key Findings, and Future Directions

This PSM model has some limitations. It uses several cut-off points that have not been validated in COVID-19 survivors but can be transferable. Neither has the PSM model been validated yet. However, we present a hierarchical model with a potential for easy implementation and benefits in primary, secondary and tertiary prevention.

COVID-19 will be with us longer than expected, with consequences not yet well established and affecting the whole population [126]. The number of COVID-19 survivors will continue to grow, leading to an increasing number of people with health needs [28,127], and it will be common to find people with different related problems. Implementing this PSM could be the start to protocolise research in the area of physiotherapy and COVID-19. In future research, the feasibility, effectiveness and cost-effectiveness of this PSM could be explored.

## 5. Conclusions

In summary, the proposed COVID19-PSM should facilitate early identification and management of long-term COVID-19 sequelae, thus responding to an arising need [6]. Work is now needed to demonstrate the reliability and cost-effectiveness of this PSM.

## Figures and Tables

**Table 1:** Search strategy used with PubMed (later adapted for use with the other databases searched).

Condition	
1	(COVID-19 [tiab]) OR (Coronavirus disease 2019[tiab]) OR (COVID *[tiab])
Cardiopulmonary system	
2	(exercise[Mesh]) OR (physical activity [tiab]) OR (physical exercise[tiab]) OR (cardiovascular system[Mesh]) OR (cardiovascular systems[tiab]) OR (circulatory system[tiab]) or (circulatory systems[tiab]) OR (cardiorespiratory fitness [Mesh]) OR (cardiorespiratory exercise testing [tiab]) OR (functional capacity [tiab]) OR (respiratory system[Mesh]) OR (respiratory systems[tiab]) OR (respiratory tract[tiab]) OR (respiratory tracts[tiab]) OR (respiratory function test[Mesh]) OR (maximal respiratory pressures[tiab]) OR (breathing exercises[tiab])
Neuromuscular system	
3	(musculoskeletal system[Mesh]) OR (musculoskeletal systems[tiab]) OR (nervous system[Mesh]) OR (neuralgia [Mesh]) OR (neuropathic pain[tiab]) OR (neuropathies[tiab]) OR (myalgia[tiab]) OR (paresthesia[Mesh]) OR (neurologic manifestation[Mesh])
Mental health	
4	(mental health [Mesh terms]) OR (mental hygiene[tiab]).
Definitive search	
5	1 AND 2
6	1 AND 3
7	1 AND 4

## Data Availability

Not applicable.

## Supplementary Materials

The following are available online at https://www.mdpi.com/article/10.3390/jcm10081691/s1: Supplementary S1. Levels of evidence of studies used in the prospective surveillance model evaluated with the Oxford Centre for Evidence-Based Medicine Level of Evidence Tool [15].
