# Supplementary material for: A COVID-19 Rehabilitation Prospective Surveillance Model for Use by Physiotherapists
Source: J Clin Med. 2021 Apr 14;10(8):1691. doi: 10.3390/jcm10081691 (PMC8071011; doi:10.3390/jcm10081691)
Supplement: Supplementary file 1 [file jcm-10-01691-s001.pdf]

Supplementary S1. Levels of evidence of studies used in the prospective surveillance model evaluated with the Oxford Centre for Evidence-Based Medicine Level of Evidence Tool [15].

**References**	**LEVEL OF EVIDENCE**
Ali NA, O'Brien JM Jr, Hoffmann SP, et al. Acquired weakness, handgrip strength, and mortality in critically ill patients. Am J Respir Crit Care Med. 2008;178(3):261-268. doi:10.1164/rccm.200712-1829OC	3
Akyuz G, Kenis O. Physiotherapy modalities and rehabilitation techniques in the management of neuropathic pain. Am J Phys Med Rehabil. 2014;93(3):253-259. doi:10.1097/PHM.0000000000000037	5
Arribas-Romano A, Fernández-Carnero J, Molina-Rueda F, Angulo-Diaz-Parreño S, Navarro-Santana MJ. Efficacy of Physiotherapy on Nociceptive Pain Processing Alterations in Patients with Chronic Musculoskeletal Pain: A Systematic Review and Meta-analysis [published online ahead of print, 2020 Feb 25]. Pain Med. 2020;pnz366. doi:10.1093/pm/pnz366	1
Australian Department of Health. Australia's Physical Activity and Sedentary Behaviour Guidelines and the Australian 24-Hour Movement Guidelines 2019 for Adulst (18-64 years). Available from: https://www1.health.gov.au/internet/main/publishing.nsf/Content/health-pubhlth-strateg-phys-act-guidelines#npa1864. Accessed on 1st May 2020	1
Barreiro E, Bustamante V, Cejudo P, et al. Guidelines for the evaluation and treatment of muscle dysfunction in patients with chronic obstructive pulmonary disease. Arch Bronconeumol. 2015;51:384-395. doi:10.1016/j.arbres.2015.04.011	5
Bennett M. The LANSS Pain Scale: the Leeds assessment of neuropathic symptoms and signs. Pain. 2001;92:147-157. doi:10.1016/s0304-3959(00)00482-6	3
Bienvenu OJ, Williams JB, Yang A, Hopkins RO, Needham DM. Posttraumatic stress disorder in survivors of acute lung injury: evaluating the Impact of Event Scale-Revised. Chest. 2013;144(1):24-31. doi:10.1378/chest.12-0908	3
Boonstra AM, Stewart RE, Köke AJ, et al. Cut-Off Points for Mild, Moderate, and Severe Pain on the Numeric Rating Scale for Pain in Patients with Chronic	3

Musculoskeletal Pain: Variability and Influence of Sex and Catastrophizing. Front Psychol. 2016;7:1466. Published 2016 Sep 30. doi:10.3389/fpsyg.2016.01466	
Cesari M, Kritchevsky SB, Penninx BW, et al. Prognostic value of usual gait speed in well-functioning older people--results from the Health, Aging and Body Composition Study. J Am Geriatr Soc. 2005;53(10):1675-1680. doi:10.1111/j.1532-5415.2005.53501.x	3
Chen YC, Chen KC, Lu LH, Wu YL, Lai TJ, Wang CH. Validating the 6-minute walk test as an indicator of recovery in patients undergoing cardiac surgery: A prospective cohort study. Medicine (Baltimore). 2018;97(42):e12925. doi:10.1097/MD.0000000000012925	3
Dettling-Ihnenfeldt DS. The Post-Intensive Care Syndrome (PICS): Impact of ICU-stay on functioning and implications for rehabilitation care. 2017	4
Ekelund U, Steene-Johannessen J, Brown WJ, et al. Does physical activity attenuate, or even eliminate, the detrimental association of sitting time with mortality? A harmonised meta-analysis of data from more than 1 million men and women [published correction appears in Lancet. 2016 Sep 24;388(10051):e6]. Lancet. 2016;388(10051):1302-1310. doi:10.1016/S0140-6736(16)30370-1	1
Elliott D, Denehy L, Berney S, Alison JA. Assessing physical function and activity for survivors of a critical illness: a review of instruments. Aust Crit Care. 2011;24(3):155-166. doi:10.1016/j.aucc.2011.05.002	5
Finnerup NB, Haroutounian S, Kamerman P, et al. Neuropathic pain: an updated grading system for research and clinical practice. Pain. 2016;157(8):1599-1606. doi:10.1097/j.pain.0000000000000492	2
Frontera WR, Slovik DM, Dawson DM. *Exercise in Rehabilitation Medicine. 2nd ed*; 2006.	5
Holland AE, Spruit MA, Troosters T, et al. An official European Respiratory Society/American Thoracic Society technical standard: field walking tests in chronic respiratory disease. Eur Respir J. 2014;44(6):1428-1446. doi:10.1183/09031936.00150314	5
Ingle L, Cleland JG, Clark AL. The long-term prognostic significance of 6-minute walk test distance in patients with chronic heart failure. Biomed Res Int. 2014;2014:505969. doi:10.1155/2014/505969	3

Jutte JE, Needham DM, Pfoh ER, Bienvenu OJ. Psychometric evaluation of the Hospital Anxiety and Depression Scale 3 months after acute lung injury. J Crit Care. 2015;30(4):793-798. doi:10.1016/j.jcrc.2015.04.006	3
Laisné F, Lecomte C, Corbière M. Biopsychosocial predictors of prognosis in musculoskeletal disorders: a systematic review of the literature (corrected and republished) *. Disabil Rehabil. 2012;34(22):1912-1941. doi:10.3109/09638288.2012.729362	1
Liu K, Zhang W, Yang Y, Zhang J, Li Y, Chen Y. Respiratory rehabilitation in elderly patients with COVID-19: A randomized controlled study. Complement Ther Clin Pract. 2020;39:101166. doi:10.1016/j.ctcp.2020.101166	2
Malfliet A, Kregel J, Meeus M, et al. Applying contemporary neuroscience in exercise interventions for chronic spinal pain: treatment protocol. Braz J Phys Ther. 2017;21(5):378-387. doi:10.1016/j.bjpt.2017.06.019	2
Mahoney FI, Barthel DW. Functional Evaluation: The Barthel Index. Md State Med J. 1965;14:61-65.	2
Mayer TG, Neblett R, Cohen H, et al. The development and psychometric validation of the central sensitization inventory. Pain Pract. 2012;12(4):276-285. doi:10.1111/j.1533-2500.2011.00493.x	2
Mo X, Jian W, Su Z, et al. Abnormal pulmonary function in COVID-19 patients at time of hospital discharge [published online ahead of print, 2020 May 12]. Eur Respir J. 2020;2001217. doi:10.1183/13993003.01217-2020	5
Nasreddine ZS, Phillips NA, Bédirian V, et al. The Montreal Cognitive Assessment, MoCA: a brief screening tool for mild cognitive impairment [published correction appears in J Am Geriatr Soc. 2019 Sep;67(9):1991]. J Am Geriatr Soc. 2005;53(4):695-699. doi:10.1111/j.1532-5415.2005.53221.x	2
Norkin CC, White DJ. *Measurement of Joint Motion : A Guide to Goniometry. 5th edition;*2017.	5
Pellegrino R, Viegi G, Brusasco V, et al. Interpretative strategies for lung function tests. Eur Respir J. 2005;26(5):948-968. doi:10.1183/09031936.05.00035205	5
Qiu S, Cai X, Sun Z, et al. Heart Rate Recovery and Risk of Cardiovascular Events and All-Cause Mortality: A Meta-Analysis of Prospective Cohort Studies. J Am Heart Assoc. 2017;6(5):e005505. Published 2017 May 9. doi:10.1161/JAHA.117.005505	2

Ramirez-Velez R, Perez-Sousa MA, Venegas-Sanabria LC, et al. Normative Values for the Short Physical Performance Battery (SPPB) and Their Association With Anthropometric Variables in Older Colombian Adults. The SABE Study, 2015. Front Med. 2020;7:52. doi:10.3389/fmed.2020.00052	2
Hunter J, Rawlings-Anderson K. Respiratory assessment. Nurs Stand. 2008;22(41):41-43. doi:10.7748/ns2008.06.22.41.41.c6576	5
Rubio Castañeda FJ, Tomás Aznar C, Muro Baquero C. Validity, Reliability and Associated Factors of the International Physical Activity Questionnaire Adapted to Elderly (IPAQ-E)]. Rev Esp Salud Publica. 2017;91:e201701004. Published 2017 Jan 18.	2
Santos GM, Souza AC, Virtuoso JF, Tavares GM, Mazo GZ. Predictive values at risk of falling in physically active and no active elderly with Berg Balance Scale. Rev Bras Fisioter. 2011;15(2):95-101. doi:10.1590/s1413-35552011000200003	3
Sidiras G, Patsaki I, Karatzanos E, et al. Long term follow-up of quality of life and functional ability in patients with ICU acquired Weakness - A post hoc analysis. J Crit Care. 2019;53:223-230. doi:10.1016/j.jcrc.2019.06.022	3
Smith JM, Lee AC, Zeleznik H, et al. Home and Community-Based Physical Therapist Management of Adults With Post-Intensive Care Syndrome [published online ahead of print, 2020 Apr 13]. Phys Ther. 2020;pzaa059. doi:10.1093/ptj/pzaa059	5
Vasconcelos KS, Dias JM, Bastone Ade C, et al. Handgrip Strength Cutoff Points to Identify Mobility Limitation in Community-dwelling Older People and Associated Factors. J Nutr Health Aging. 2016;20(3):306-315. doi:10.1007/s12603-015-0584-y	4
World Health Organization. Waist circumference and waist-hip ratio: report of a WHO expert consultation. Geneva, 8-11 December 2008. Accessed at https://apps.who.int/iris/bitstream/handle/10665/44583/9789241501491_eng.pdf?ua=1 on May 5, 2020.	1
Yang LL, Yang T. Pulmonary Rehabilitation for Patients with Coronavirus Disease 2019 (COVID-19) [published online ahead of print, 2020 May 14]. Chronic Dis Transl Med. 2020;10.1016/j.cdtm.2020.05.002. doi:10.1016/j.cdtm.2020.05.002	5
Zhao HM, Xie YX, Wang C. Recommendations for respiratory rehabilitation in adults with COVID-19 [published online ahead of print, 2020 Apr 9]. Chin Med J (Engl). 2020;10.1097/CM9.0000000000000848. doi:10.1097/CM9.0000000000000848	5
